# Cardiac rehabilitation: Effective yet underutilized in patients with cardiovascular disease

**DOI:** 10.1002/clc.23911

**Published:** 2022-09-02

**Authors:** Natalie J. Bracewell, Jeffrey Plasschaert, Charles Richard Conti, Ellen C. Keeley, Jamie B. Conti

**Affiliations:** ^1^ Department of Medicine University of Florida Gainesville Florida USA; ^2^ Division of Cardiovascular Medicine University of Florida Gainesville Florida USA

**Keywords:** cardiac rehabilitation, chronic heart failure, ischemic heart disease, peripheral arterial disease, structured exercise

## Abstract

Cardiac rehabilitation is a comprehensive program that treats patients with multiple cardiac conditions including post‐myocardial infarction, stable angina, post‐coronary artery bypass surgery, chronic heart failure, and peripheral vascular disease with structured exercise, and nutrition and risk factor counseling. It is an effective tool that has been shown to improve not only quality of life but also reduce adverse cardiac events, including death. While the value of cardiac rehabilitation is supported by a large body of evidence and its recommendation by the American Heart Association/American College of Cardiology it is significantly underutilized due to both patient and systemic factors. Continued efforts should be made to remove the obstacles to make cardiac rehabilitation available to all those who qualify.

## 1 INTRODUCTION

Cardiac rehabilitation is a comprehensive program that includes an individualized exercise plan along with counseling on management and reduction of risk factors for cardiovascular disease. These include nutritional counseling and weight management, discussion of blood pressure control, diabetes and lipid management, tobacco cessation, psychosocial assessment and counseling on physical activity.^1^ Cardiac rehabilitation has been shown to improve mortality in patients with heart failure and coronary artery disease as well as reduce risk factors for cardiovascular disease and improve quality of life.^2^, ^3^, ^4^

Because of these proven benefits, cardiac rehabilitation is highly recommended by the American Heart Association and American College of Cardiology (AHA/ACC). Cardiac rehabilitation is a Class 1 indication for patients with acute coronary syndromes (ACS), as well as non‐ST elevation and ST elevation myocardial infarctions (MI).^5^, ^6^ It is also a Class 1 recommendation for patients postrevascularization in the non‐ACS setting,^7^ and a Class 2a recommendation for patients with stage C heart failure.^8^ Moreover, a supervised exercise program, including cardiac rehabilitation, is also a Class 1 recommendation for patients with peripheral arterial disease.^9^

## 2 HISTORY

Before the 1950s, the general approach to the management of myocardial infarction was bed rest and physical inactivity. A change in this method of management started with the recommendation of cardiac chair therapy, which involved patients sitting in a chair for a 1−2 h per day.^10^ Over the following years, the recommendations included progressively more physical activity and in the 1970s a structured and supervised rehabilitation program was established.^10^ This program contained three phases. Phase I was initiated while the patient was still in the hospital after a cardiovascular event as low‐level supervised activity. This was followed by Phase II, which was supervised exercise on an outpatient basis with ECG monitoring. Next was Phase III, a community‐based or gymnasium rehabilitation plan where patients may or may not have had medical monitoring.^11^

**Figure 1:**
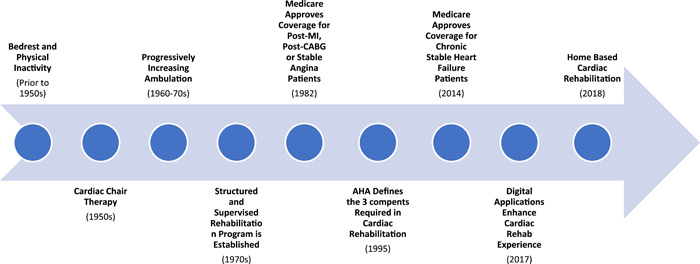
Cardiac rehabilitation timeline

The American Heart Association published a statement in 1995 that delineated the required components of any cardiac rehabilitation program: (1) training in exercise with prescribed activity, (2) modification of risk factors, and (3) psychosocial assessment and counseling.^12^ In 1982, Medicare began to provide insurance coverage for cardiac rehabilitation in patients who were enrolled within one year of an acute MI, status postcoronary artery bypass graft (CABG) surgery, or had been experiencing chronic stable angina.^13^ In 2014 Medicare expanded its coverage for cardiac rehabilitation to patients with chronic, stable heart failure who had not had a recent (within 6 weeks) hospitalization.^14^ Cardiac rehabilitation structure and indications have evolved over the years and there continues to be important and significant change. (Figure 1).

### 2.1 Coronary artery disease

The first group of patients in whom cardiac rehabilitation was intended was those who had sustained an acute MI, were post‐CABG or had stable angina.^12^ In the 2021 Cochrane Review, “Exercise‐based cardiac rehabilitation for coronary heart disease,” investigators analyzed 85 trials enrolling a total of 23 430 patients with coronary artery disease. They found significantly lower rates of MI and all‐cause hospitalization at 6−12 months. Moreover, at medium‐term follow‐up (>12−36 months), and long‐term follow‐up (>36 months) there were lower rates of MI and cardiovascular mortality in patients who had undergone cardiac rehabilitation. Lastly, at 1 year follow‐up, patients who participated in cardiac rehabilitation reported a significant improvement in health‐related quality of life.^15^

A randomized controlled trial of 101 men with single vessel coronary artery disease with stenosis ≥75% and Class 1−3 angina symptoms (using the Canadian Cardiovascular Society [CCS] classification system) with ischemia present on treadmill or nuclear stress test were randomized to percutaneous coronary intervention (PCI) or exercise training and followed for 1 year.^16^ There was significant improvement in clinical symptoms noted by the drop in CCS score from baseline to 12 month follow‐up in both the PCI (1.7 vs. 0.7, *p* < .001) and structured exercise (1.5 vs. 0.4, *p* < .001) groups from baseline. On follow‐up, patients randomized to the exercise group experienced a 20% increase in maximal exercise tolerance (133−159 W, *p* < .001) while those randomized to PCI had no change in exercise tolerance of 130 W before and after cardiac rehab. The ischemic threshold (time to signs of ischemia during stress testing including ST depressions and/or angina), increased by 30% in the exercise group (133−159 W, *p* < .001) and 16% in the PCI group (99−119 W, *p* < .05). At 12 month follow‐up, the event‐free survival was significantly higher in those randomized to exercise compared to those randomized to PCI (88% vs. 70%, *p* = .023). Major adverse cardiac events included stroke, target‐vessel revascularization, PCI or CABG. This study supports the recommendation of cardiac rehabilitation participation for patients with single‐vessel stable angina to not only improve symptoms but also for the reduction of other adverse cardiovascular events.^16^

Crehabilitation have also been documented in elderly patients with coronary artery disease. A study evaluated 601 099 Medicare patients ≥ 65 years from 1997 through 2002 who were hospitalized for coronary artery disease treated with PCI or CABG.^2^ Investigators found significantly lower annual mortality in those who participated in cardiac rehabilitation compared to those who did not at each year (2.2% vs. 5.3%, 5.2% vs. 10.1%, 8.5% vs. 14.8%, 12.2% vs. 19.6%, 16.3% vs. 24.6%, all *p* < .001). Of the patients who attended cardiac rehabilitation, “high‐users” (those who attended ≥25 sessions), had significantly lower 1 year mortality (1.1% vs. 2.6%, *p* < .011) and 5 year mortality (14% vs. 17.2%, *p* < .011) compared to “low‐users” (those who attended ≤24 or less sessions).^2^

### 2.2 Heart failure

Results from the HF‐ACTION (Heart Failure: A Controlled Trial Investigating Outcomes of Exercise Training) trial supports cardiac rehabilitation in the heart failure population.^17^ This study was a multicenter, randomized, controlled trial that enrolled 2331 patients from 2003 to 2007. Enrollment criteria included patients with an ejection fraction ≤35% who had New York Heart Association (NYHA) Class 2−4 symptoms. Participants were randomized to an exercise protocol with structured and supervised exercise sessions 3 days per week for a total of 36 sessions or usual medical care with no structured exercise program. There was a reduction in the primary outcome (cardiovascular mortality or heart failure hospitalization) for the exercise training group versus usual care group after adjusting for confounding factors (30% vs. 34%, *p* = .03). In addition, in the exercise versus control group there was a significant increased distance in the 6‐min walk test from baseline to 3 months (20 m vs. 5 m, *p* < .001), increase in cardiopulmonary exercise time at 3 months (1.5 min vs. 0.3 min, *p* < .001) and 12 months (1.5 min vs. 0.2 min, *p* < .001), and increased peak oxygen consumption at 3 months (0.6 vs. 0.2 ml/kg/min) and 12 months (0.7 vs. 0.1 ml/kg/min, *p* < .001).^17^

A meta‐analysis including 13 studies (3990 patients) with heart failure evaluated the impact of participation in cardiac rehabilitation on quality of life and exercise capacity.^18^ The majority of participants (97%) had heart failure with reduced ejection fraction. The Minnesota Living with Heart Failure Questionnaire was used to measure the health‐related quality of life and showed a significant improvement in the patients who had undergone cardiac rehabilitation (mean improvement 5.9, *p* = .018). In addition, assessment using the 6‐min walk test showed a significant improvement in patients who also had participated in cardiac rehabilitation (mean difference 21.0 m, *p* = .034).^18^

### 2.3 Peripheral arterial disease

Cardiac rehabilitation not only helps with secondary risk reduction in subjects with peripheral arterial disease, many of whom also have coronary artery disease, but also improves physical mobility. A study by Perkins and colleagues evaluated 56 patients with stable claudication symptoms who were randomized to treatment with percutaneous revascularization versus exercise training.^19^ The patients were followed at 6, 9, 12, and 15 months. The distance to claudication symptoms was significantly lower in patients who were randomized to the exercise training compared to percutaneous revascularization at 6 months (*p* = .005) up to 15 months (*p* = .0001). Interestingly, there were significant improvements in the arterial brachial pressure indices (ABPI) levels in those who were randomized to percutaneous revascularization compared to those who were randomized to exercise training (*p* ≤ .04). This lack of ABPI change despite increased walking distance in the structured exercise training group is likely due to changes at the cellular level that improve oxygen utilization by tissues.^19^

A Cochrane review including 21 trials (1400 subjects) compared three different modalities of exercise for patients with peripheral arterial disease: supervised exercise therapy, home‐based exercise therapy, and walking.^20^ Investigators found that there was increased pain‐free walking distance of 120 meters after 3 months in patients who had undergone the supervised exercise therapy (standard median difference 0.51, *p* = .0009). They also found an increased maximal walking distance of 210 meters in patients who had undergone supervised exercise therapy versus walking advice at 3 months (standard mean difference 0.8, *p* = .00001). There was also a longer pain‐free walking distance and improved quality of life in the structured exercise therapy group compared to the home‐based exercise therapy group and walking group.^20^ Structured exercise benefits patients with symptomatic peripheral arterial disease through improvement in walking distance without symptoms in addition to providing guidance on diet and smoking cessation counseling.

### 2.4 Geriatric and frail population

The geriatric and frail population is one that may have been determined as too high‐risk to undergo cardiac rehabilitation. Nonetheless, studies have shown cardiac rehabilitation to be beneficial in this group.

In one study investigators evaluated changes in risk cardiac factors including cholesterol, body mass index (BMI), body fat percentage and exercise capacity as well as different aspects of behavior and quality of life.^4^ They separated the patients into elderly (≥65 years old) and young (<65 years old) categories. There were statistically significant reductions in body fat percentage (25.4% vs. 23.7%, *p* < .0001) and BMI (25.8 vs. 25.6 kg/m^2^, *p* < .01), and increase in exercise capacity (5.4 metabolic equivalents (METs) vs. 7.7 METs, *p* < .0001) in elderly patients comparing before to after cardiac rehabilitation. Significant improvement was also seen using scoring systems in levels of anxiety (4.2 vs. 3.0, *p* < .01), depression (3.3 vs. 4.0, *p* < .01), somatization (6.2 vs. 4.0, *p* < .0001), mental health (23.6 vs. 24.8, *p* < .0001), energy (14.7 vs. 17.2, *p* < .0001) and overall well‐being (46.5 vs. 51.8, *p* < .0001) in this elderly population comparing before to after cardiac rehabilitation.^4^

Clinical improvements in elderly patients with heart failure who undergo cardiac rehabilitation have also been reported. The REHAB‐HF (Rehabilitation Therapy in Older Acute Heart Failure Patients) study was a prospective, multicenter randomized controlled trial that evaluated patients ≥60 years old who had been hospitalized with acute decompensated heart failure and were then randomized to either cardiac rehabilitation or usual care.^21^ The primary outcome was the Short Physical Performance Battery which consists of gait speed, strength (through a sit‐to‐stand test) and a standing balance test. While baseline scores were similar, patients who participated in cardiac rehabilitation had significant improvement in the Short Physical Performance Battery at 3 months compared to those who had usual care (8.3 vs. 6.9, *p* < .001).^21^

Frailty, however, is a factor that may dissuade providers from referring patients to cardiac rehabilitation. This issue was studied by Kamiya  et al.^3^ in a multicenter retrospective cohort study 3277 patients with heart failure from 15 hospitals in Japan were enrolled to compare rates of all‐cause mortality and heart failure hospitalization in patients who had participated in cardiac rehabilitation versus those who had not. They found a significantly lower rate of the composite outcome of all‐cause mortality and heart failure hospitalization in those who participated in cardiac rehabilitation compared to those who did not (Hazard Ratio [HR] 0.77 [95% confidence interval [CI]: 0.65−0.92], *p* = .003). Secondary outcomes were also lower in patients who had undergone cardiac rehabilitation including all‐cause mortality (HR 0.67 [95% CI: 0.51−0.87], *p* = .003) and heart failure hospitalizations (HR 0.82 [95% CI: 0.67−0.99], *p* = .044). Investigators then further separated patients based on their Frailty Index (FI). The FI was calculated by the presence of risk factors (hypertension, diabetes mellitus, coronary artery disease, cerebrovascular disease, atrial fibrillation, dyslipidemia, hyperuricemia, dementia, cancer, myocardial infarction, or prior heart failure hospitalization) as well as anemia, hypoalbuminemia, renal dysfunction, obesity, low BMI, and age >75 years old, need for assistance with activities of daily living and mobility limitation. The number of risk factors for each patient were summed and divided by 19 to create the index. Patients were then divided into groups according to the extent of frailty including those who were deemed fit (FI < 0.21), mild frailty (FI = 0.21−0.31), moderate frailty (FI = 0.32−0.41) and severe frailty (FI ≥ 0.42) and assessed for the primary outcomes in patients who did and did not participate in cardiac rehabilitation. There were significantly lower rates of the composite endpoint (all‐cause mortality and heart failure hospitalizations) for the patients who had undergone cardiac rehabilitation in the categories of fit (Adjusted HR: 0.55 [95% CI: 0.39−0.77], *p* = .001), mild frailty (Adjusted HR: 0.62 [CI: 0.47−0.82], *p* = .001) and moderate frailty (Adjusted HR: 0.69 [95% CI: 0.48−0.98], *p* = .038). The only group that did not benefit from cardiac rehabilitation was those with severe frailty (Adjusted HR: 0.83 [95% CI: 0.65−1.06], *p* = .127).^3^

### 2.5 Current state

Despite convincing evidence for improved morbidity and mortality and ACC/AHA guideline recommendations, cardiac rehabilitation is grossly underutilized. Following an acute MI only one‐third of patients participate in cardiac rehabilitation and participants are less likely to be female, black, and uneducated.^22^, ^23^ In a study of Medicare patients from 2016 to 2017, only 24.4% of patients with qualifying diagnoses attended cardiac rehabilitation.^24^ Moreover, of those who participated, only 26.9% of patients attended 36 or more sessions. There are multiple factors that contribute to the low participation and program completion of patients in cardiac rehabilitation. These include cost and travel barriers, gender disparities, racial or ethnic healthcare disparities, medical comorbidities, and low rates of physician referrals.^25^ With multiple factors limiting the participation and completion of patients in cardiac rehabilitation programs, increasing awareness of the benefits of cardiac rehabilitation may increase referrals and participation.

Starting in July 2016, Dr. C. Richard Conti developed and supervised the cardiac rehabilitation program at the University of Florida (UF). The UF program experienced rapid growth over the first few years starting with 40 patients enrolled in 2016 with 18% of patients graduating from the program. By 2018, this increased to 208 enrolled patients with a 32% graduation rate. Despite the COVID‐19 pandemic, enrollment remained high (214 patients in 2020, and 302 patients in 2021), but graduation rates decreased to 17% and 11%, respectively.

At UF we continue to promote the cardiac rehabilitation program that was started by Dr. C. Richard Conti nearly 7 years ago. For example, as part of a quality improvement project, we significantly increased the proportion of ACS patients referred to our cardiac rehabilitation program from 10% to 43% (*p* < .001) by instituting a combination of an automated referral order in the electronic medical record, participation of the cardiac rehabilitation staff on discharge rounds, and housestaff and patient education regarding the benefits of cardiac rehabilitation.^22^, ^23^ In addition, the cardiac rehabilitation gym was relocated to a more centrally‐located area adjacent to the hospital making access easier for patients.

### 2.6 The future of cardiac rehabilitation

Modifying the structure of cardiac rehabilitation, for example, using telehealth to allow participation to be done,^26^, ^27^ and using technology for metric tracking,^28^ and reminders to encourage patients throughout their exercise program^29^ are all examples of recent changes that have substantially improved access to cardiac rehabilitation.

There is new interest in comparing high‐intensity interval training (HIIT) compared to the standard moderate‐intensity continuous training (MICT) in cardiac rehabilitation programs to see if it improves patient outcomes. In one study patients with coronary artery disease were randomized into either HIIT or MICT, also known as usual care.^30^ Patients participated in three sessions/week of their assigned exercises for 2 supervised weeks then 1 home‐based week followed by an additional 11 months of home‐based exercises three times weekly. At 12 month follow‐up, patients who underwent HIIT and continued with the exercise program had increased peak mVO_2_ compared to patients in the MICT group (2.9 vs. 1.2 ml/kg/min, *p* = .02). There was no significant difference between groups in glucose tolerance, reduction in blood pressure or quality of life. There were no deaths or cardiovascular events related to the exercise in either the HIIT or MICT groups.^30^ This study supports both moderate and high intensity exercise as being beneficial in cardiac rehabilitation patients.

Advancements in technology can be used to enhance the cardiac rehabilitation experience and improve outcomes for patients. In a randomized controlled trial, ACS patients treated with PCI were randomized to standard cardiac rehabilitation or cardiac rehabilitation plus digital health intervention (DHI).^28^ The DHI curriculum included a smartphone‐ or web‐based application that required patients to enter their weight, BMI, blood pressure, laboratory values including glucose and lipids, physical activity, quality of life, adherence to medications, and smoking history. Patients were encouraged to enter these metrics throughout the duration of the cardiac rehabilitation program. Significant differences between with cardiac rehabilitation plus DHI compared to the cardiac rehabilitation only group at the 90 day follow‐up included change in weight (−5.1 vs. −0.8 kg, *p* = .02), BMI (−1.6 vs. −0.3 kg/m^2^ *p* = .01), waist circumference (−8.3 vs. 1.1 cm, *p* = .01) and body fat percentage (−4.5% vs. −1.1%, *p* = .02). There was no significant difference between the two groups with respect to blood pressure, laboratory values, depression or rehabilitation attendance.^28^

With respect to mobile technology, a study by Imran^29^ conducted a propensity score‐matched study for patients enrolled in cardiac rehabilitation where patients were given the option of cardiac rehabilitation only or a mobile technology cardiac rehabilitation group. The mobile technology group used the Wellframe mobile technology, an application that tracked patient's metrics and provided educational materials and the ability to message the cardiac rehabilitation team with questions.^29^ The patients in the cardiac rehabilitation group with the mobile technology were found to attend more cardiac rehabilitation sessions (mean 28 vs. 22 sessions *p* = .009) and had a 1.6 times more likelihood of completing cardiac rehab (*p* = .046) compared to those who did not opt for the mobile technology. In addition, there was significantly more weight loss in the cardiac rehabilitation patients who used the mobile technology (−2.1 pounds [95% CI: −3.47 to −0.47], *p* = .012).^29^

Another aspect of cardiac rehabilitation that continues to be evaluated is home‐based cardiac rehabilitation. There are many benefits to home‐based programs, especially as transportation and distance from rehabilitation centers can be limiting factors in patients' participation, in addition to concerns of infection throughout the COVID pandemic. In one randomized, controlled trial heart failure patients with an ejection fraction < 50% were randomized to a home‐based cardiac rehabilitation group versus a control group with usual medical care.^26^ The cardiac rehabilitation group participated in 1 week of outpatient rehabilitation then transitioned to home‐based rehabilitation for three, 30‐min sessions per week. At 90 day follow‐up, patients who had undergone home‐based cardiac rehabilitation had significant improvement in their peak VO_2_ (18.2 ml/kg/min before and 20.9 ml/kg/min at follow‐up, *p* = .02) and anaerobic threshold (5.5 ml/kg/min before and 6.0 ml/kg/min at follow‐up, *p* = .005). In addition, home‐based cardiac rehabilitation program subjects had improvement in their Minnesota Living with Heart Failure Questionnaire scores (from 32.1 to 20.2, *p* < .01).^26^ On the other hand, patients randomized to usual care had a significant reduction in their peak VO_2_ at follow‐up (from 18.7 to 16.5 ml/kg/min, *p* < .01).^26^

In a randomized control trial by Bravo‐Esocbar et al. home‐based cardiac rehabilitation was compared to hospital‐based cardiac rehabilitation for 28 patients with stable coronary artery disease.^27^ The home‐based cardiac rehabilitation group had one session of supervised exercise per week in the outpatient center and two sessions with remote electrocardiographic monitoring using a device. Those who participated in the usual cardiac rehabilitation program attended the program three times per week. Investigators found no difference in METs, BMI, lipids, A1C levels, and blood pressure between the home‐based cardiac rehabilitation group and the hospital‐based cardiac rehabilitation group. The only difference was a significant improvement in quality of life scores using the Medical Outcome Survey Short Form for patients randomized to the hospital‐based cardiac rehabilitation group compared to the home‐based group (10.3 vs. −4.31, *p* = .004).^27^ These studies suggest that home‐based cardiac rehabilitation may be as effective in improving patient's exercise capacities and, from a practical standpoint, are safe alternatives with respect to COVID infection.

## 3 CONCLUSION

Cardiac rehabilitation has been shown to effectively reduce mortality, modify risk factors and enhance quality of life, and therefore is a Class 1 indication for multiple cardiac dignoses.^5^, ^6^, ^7^, ^8^, ^9^ Despite its proven benefits, cardiac rehabilitation continues to be an underutilized resource, which is secondary to multiple patient and institutional factors. Initiatives to educate patients and physicians about the short‐ and long‐term health benefits of cardiac rehabilitation, increase referrals of patients to cardiac rehabilitation programs, augment availability of programs across the country, expand home‐based programs for underserved areas, and improve insurance coverage are crucial to make this valuable resource to the wide population of patients who could benefit from the program.

## CONFLICT OF INTEREST

The authors declare no conflict of interest.

## Data Availability

Data sources can be available on request. If the above statement is not acceptable and more specific information is needed, please use this statement: The data used in this review are available on PubMed in their original article forms. Here are the following articles by their reference number and corresponding doi numbers: doi:10.1016/s0002-9149(99)80054-x,4 doi:10.1161/01.CIR.0000121360.31954.1.F,16 doi:10.1016/j.jacc.2009.01.078,17 doi:10.1001/jama.2009.454,18 doi:10.1002/ejhf.1311,19 doi:10.1016/s1078-5884(96)80171-7,20 doi:10.1002/14651858.CD005263.pub4,21 doi:10.1056/NEJMoa2026141,22 doi:10.1161/CIRCHEARTFAILURE.119.006798,23 doi:10.1161/JAHA.117.007664,24 doi:10.1097/HPC.0000000000000263,25 doi:10.1161/CIRCOUTCOMES.119.005902,26 doi:10.1001/jamacardio.2020.3511,28 doi:10.1016/j.ahj.2017.02.016,29 doi:10.1161/JAHA.120.020482,30 doi:10.1097/MD.0000000000009629,31 doi:10.1186/s12872-017-0499-0.32.
